# Fungal Diseases in Taiwan—National Insurance Data and Estimation

**DOI:** 10.3390/jof5030078

**Published:** 2019-08-21

**Authors:** Yu-Shan Huang, David W. Denning, Shu-Man Shih, Chao A. Hsiung, Un-In Wu, Hsin-Yun Sun, Pao-Yu Chen, Yee-Chun Chen, Shan-Chwen Chang

**Affiliations:** 1 Department of Internal Medicine, National Taiwan University Hospital, Taipei 10002, Taiwan; 2 National Aspergillosis Center, Wythenshawe Hospital, Manchester M23 9LT, UK; 3 The University of Manchester and Manchester Academic Health Science Centre, Manchester M13 9PL, UK; 4 Institute of Population Health Sciences, National Health Research Institutes, Zhunan, Miaoli County 35053, Taiwan; 5 Department of Medicine, National Taiwan University College of Medicine, Taipei 10051, Taiwan; 6 National Institute of Infectious Diseases and Vaccinology, National Health Research Institutes, Zhunan, Miaoli County 35053, Taiwan

**Keywords:** fungal infections, epidemiology, nationwide estimation, Taiwan

## Abstract

The burden of fungal diseases based on the real-world national data is limited. This study aimed to estimate the Taiwan incident cases with selected fungal diseases in 2013 using the National Health Insurance Research Database (NHIRD) which covered 99.6% of the 23.4 million population. Over 80,000 incident cases were found and the majority were superficial infections including vulvovaginal candidiasis (477 per 100,000 adult women) and oral candidiasis (90 cases per 100,000 population). Common potentially life-threating fungal diseases were *Pneumocystis* pneumonia (5.35 cases per 100,000 population), candidemia (3.68), aspergillosis (2.43) and cryptococcal meningitis (1.04). Of the aforementioned cases cancer patients contributed 30.2%, 42.9%, 38.6% and 22.2%, respectively. Of 22,270 HIV-infected persons in NHIRD in 2013, four common diseases were *Pneumocystis* pneumonia (28.3 cases per 1000 HIV-infected patients), oral candidiasis (17.6), esophageal candidiasis (6.06) and cryptococcal meningitis (2.29). Of pulmonary aspergillosis 32.9% occurred in patients with chronic pulmonary diseases and 26.3% had a prior diagnosis of tuberculosis. There are some notable gaps related to insurance claim data. Cutaneous, urinary tract and eye fungal infections were not captured.

## 1. Introduction

Burdens of fungal disease vary widely by pathogen, types of infection, patient populations and country/region [1]. The spectrum of fungal diseases ranges from superficial, noninvasive to deep and disseminated infection. Pathogens causing superficial infections in healthy individuals may result in invasive fungal diseases in patients with hematologic malignancies, HIV infection, neonates, or the elderly, which are associated with significant morbidity and mortality [2,3].

Fungal pathogens are virtually ignored by the press, the public and funding bodies, despite posing a significant threat to public health as well as food biosecurity and biodiversity [4]. One major reason is limited available epidemiological data of high quality or representative of disease burden. A precise estimation of disease burden is crucial for decision making regarding resource allocation and development of preventive, diagnostic, and therapeutic strategies. With the advances in medical care and the progress in treatment of hemato-oncologic and rheumatologic diseases, the population of immunosuppressed patients is expanding, and an increase in the number of fungal diseases has been observed [2,5]. On the other hand, with the encouragement of rapid initiation of anti-retroviral therapy (ART) among HIV-infected patients in the resource-rich areas, the number of opportunistic fungal infections such as *Pneumocystis* pneumonia (PCP), disseminated cryptococcosis, or talaromycosis (formerly penicilliosis) are decreasing in patients with HIV/AIDS [6,7]. Due to changing medical practices, the epidemiologic trend of each fungal disease in different host populations is expected to be distinct [8].

High quality epidemiological studies of infectious diseases depend on the alertness and awareness of both patients and physicians and the ready availability of diagnostic tools which are implemented in a timely fashion. This is often a major barrier to conducting epidemiological studies of fungal infections. Thus, studies of severe fungal diseases are mostly limited to developed countries or tertiary care centers. Therefore, there is a concern of bias generated during a systemic review based on current publications. Nationwide active surveillance is considered the most comprehensive approach to evaluate the disease burden, but is demanding of resources and data are usually not available in real time. 

Since the National Health Insurance in Taiwan was launched in 1995 and covers almost all the population, its data may offer valuable information in this regard. This is the first study aimed to comprehensively estimate the burden of various fungal diseases at country level in the whole population as well as in different patient groups using the Health Insurance Research Database (NHIRD), and compare the incidence and prevalence of fungal diseases with nearby Asian countries.

## 2. Methods and Materials

### 2.1. Study Design and Population

This nationwide, population-based, cross-sectional study aimed to estimate annual incident cases and incidence rates of various fungal diseases in 2013 in Taiwan. The study cohort consisted of patients randomly selected with a one-in-three sampling ratio from the total population (inpatient and outpatient settings) registered from 1 January 2013 through 31 December 2013 in NHIRD. 

Taiwan has an estimated population of 23.4 million people in 2013, 20% are children and 47% are adults over 40 years old. The gross domestic product (GDP) per capita was 25,026 USD in 2013 [9].

The study was approved by the Research Ethics Committee of National Taiwan University Hospital, and written or oral informed consent was waived (registration number 201511026RINB, approved on 30 November 2015).

### 2.2. Database

The National Health Insurance of Taiwan is a nationwide compulsory healthcare program that covers an estimated 99.6% of the population [10] and contracts with more than 90% of Taiwan’s healthcare providers in hospitals and clinics. To ensure service quality and to avoid unnecessary expenses, after all claims are reimbursed, the National Health Insurance Administration retrospectively checks the appropriateness of medical claims by random peer review. 

The NHIRD used in this study consists of de-identified secondary data released for research purposes and includes information of hospital admissions, outpatient visits, disease profiles, prescriptions, interventional procedures. Diseases are coded based on the International Classification of Disease, Ninth Revision, Clinical Modification (ICD-9-CM) code. 

For outpatients with multiple diseases, a maximum of four diseases are provided; whereas the maximum is five for inpatients. The rationale of selecting diagnoses or procedures is mainly driven by reimbursement purposes. For patients who are cared for in more than one hospital service during the index hospitalization, only the data from the hospital service before discharge was provided. 

### 2.3. Patients and Definitions

Patients with newly diagnosed fungal diseases (incident cases) in 2013 were analyzed. Information of each patient was extracted from the dataset from 1 January 2012 to 31 December 2013. Therefore, there was at least one-year of data before enrollment to include and verify all underlying diseases or comorbidities of each patient and exclude patients whose fungal diseases were diagnosed before 2013. Each fungal disease was identified with the ICD-9 code as listed in Table 1. A case of a selected fungal disease was defined as coding with a specific ICD-9 code at discharge of the index hospitalization or at least twice in the outpatient record in 2013. Patients were categorized into five groups according to their underlying diseases, *c*omorbidities or clinical conditions, all data extracted within the preceding six months prior to the first diagnosis of a fungal disease: HIV-infection, chronic pulmonary disease, cancer (including hematological malignancies and solid tumors), admission to intensive care units (ICU), or without underlying *c*omorbidities listed in Supplementary Table S1. The *c*omorbidities were defined based on ICD-9-CM coding algorithms for Charlson comorbidities (Supplementary Material 1) [11]. Comorbidities that were coded in at least two outpatient visits or one discharge diagnosis for inpatients within six months preceding the index date were included for analysis. The index date was the date of diagnosis of a fungal disease, which corresponds to the date of index admission or the first diagnosis in outpatient records. 

Immunosuppressive therapies included in the analysis were systemic corticosteroid, immunosuppressant and anti-neoplastic agents. Exposure to these drugs was defined as the use of medications with the ATC code H02AB (glucocorticoids), L04A (immunosuppressant), and L01 (anti-neoplastic agent) in the proceeding one month prior to the index date in the database. 

### 2.4. Data Analysis

The Microsoft SQL Server 2008 R2 (Microsoft Corporation, Redmond, WA, USA) was used for data linkage, processing, and sampling. The annual incidence rate of each fungal disease was presented as an incident case number per 100,000 populations (whole population in the NHIRD) or per 100,000 patients (selected patient groups). 

## 3. Results 

**Figure 1:**
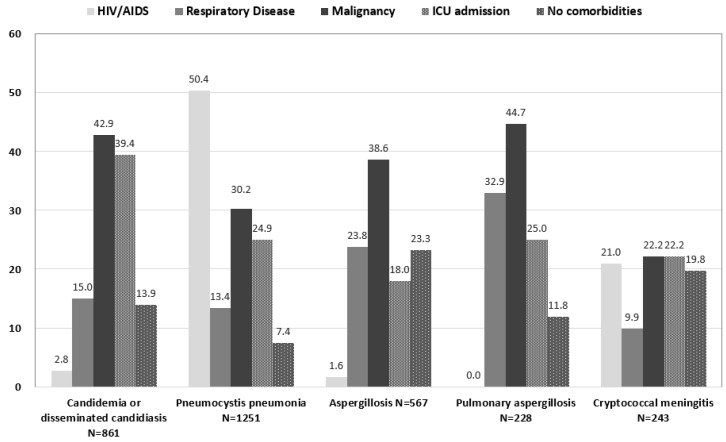
The proportion of selected patient groups among patients with potentially life threating fungal diseases, Taiwan, 2013. The group “Aspergillosis” included the diagnosis code 117.3 and 484.6; the group “Pulmonary aspergillosis” included the diagnosis code 484.6. The subgroup “No comorbidities” includes patients without underlying diseases listed in Charlson comorbidities [11]. Abbreviations: AIDS, acquired immune deficiency syndrome; HIV, human immunodeficiency virus; ICU, intensive care unit.

Table 1 summarizes the estimates for the incident case numbers and incidence rates of selected fungal diseases for the whole population in 2013 in Taiwan. Over 80,000 incident cases were found and the majority of them were superficial infections caused by *Candida* species. There included 45,291 cases of vulvovaginal candidiasis (477 per 100,000 adult women) and 21,066 cases of oral candidiasis or oral thrush (90 cases per 100,000 population). The leading four life-threating fungal diseases and their incidence rates were PCP (1251 cases, 5.35 cases per 100,000 population), systemic or disseminated candidiasis (861 cases, 3.68 cases per 100,000 population), aspergillosis (567 cases, 2.43 cases per 100,000 population), and cryptococcal meningitis (1.04 cases per 100,000 population). The incident case numbers and the proportions of the selected patient group among patients with potentially life threating fungal disease were shown in Figure 1.

There were 22,270 HIV-infected persons in NHIRD in 2013. Therefore, the most common five fungal diseases and their incidence rates were PCP (28.3 cases per 1000 HIV-infected patients), oral candidiasis or oral thrush (17.6 cases per 1000 patients), esophageal candidiasis (6.06 cases per 1000 patients), cryptococcal meningitis (2.29 cases per 1000 patients), systemic candidiasis (1.1 cases per 1000 patients) and aspergillosis (0.4 cases per 1000 patients). These incidences were much higher than those for the whole population. 

### 3.1. Candidiasis

Among various fungal diseases, candidiasis was the most common disease. Of 45,291 cases of vulvovaginal candidiasis 9,363 cases had a recurrence of four episodes or more in 2013 (98 per 100,000 adult women). Of the 861 cases of systemic candidiasis, 42.9% were cancer patients and 39.4% were ICU patients. 

### 3.2. Pneumocystis Pneumonia

Of 1251 cases with PCP, 50.4% were HIV-infected patients and 24.9% patients discharged from the ICU, Figure 1. Of 378 cancer patients, 273 cases were reported from those who had received immunosuppressive therapy during the index hospitalization course and 105 of those without immunosuppressive therapy.

### 3.3. Aspergillosis

In 2013 there were 567 cases of aspergillosis other than allergic bronchopulmonary aspergillosis (ABPA). Of them, 38.6% were cancer patients, 23.8% patients had chronic pulmonary diseases, and 18.0% occurred in ICU patients. In 132 (23.3%) patients there was no underlying disease/comorbidity, which was included in the calculation of the Charlson comorbidity index in the Supplementary Material 1. Among 567 cases of aspergillosis, 228 cases were coded as pulmonary aspergillosis. Of them, 44.7% occurred in cancer patients, 32.9% in patients with chronic pulmonary diseases. There were 60 (26.3%) patients with a prior diagnosis of tuberculosis. 

Of 18 incident cases with ABPA (incidence rate, 0.077 per 100,000 population), nine (50%) had a chronic pulmonary disease. Of note, there was a total of 45 prevalent cases with ABPA in the same year and the prevalence rate was 0.19 per 100,000 population. Among them, 24 (53.3%) patients had chronic pulmonary disease, and 12 (26.7%) cases no underlying comorbidities.

### 3.4. Cryptococcal Meningitis

There were 243 cases of cryptoccocal meningitis, 21% was HIV-infected, 22.2% had cancers, and 22.2% diagnosed in ICU patients. Of note, 19.8% did not have any underlying comorbidities. 

### 3.5. Other Fungal Diseases 

The case numbers of life-threatening fungal diseases caused by any etiology other than the aforementioned were relatively few. For example, there were only 66 mucormycosis cases (0.28/100,000 population) and 57 histoplasmosis cases (0.24/100,000 population). A small number of chromoblastomycosis, sporotrichosis, and mycetoma cases are seen each year.

## 4. Discussion

This is the first study describing the nationwide annual burden of fungal diseases and its distribution in selected patient groups in Taiwan. Over 80,000 episodes of fungal diseases were diagnosed in 2013 among 23.4 million Taiwanese. Among various fungal pathogens, candidiasis, aspergillosis, PCP and cryptococcosis were the most common potentially life threatening diseases. 

Invasive infections with *Candida* species continue to represent a major health and economic burden, and are associated with additional mortality and morbidity in an already debilitated hospital patients [15]. Candidemia is the most common form of systemic candidiasis. According to a recent survey and estimation facilitated by the Leading International Fungal Education (LIFE) portal the global incidence of invasive candidiasis has been estimated at 750,000 cases annually (1.8 to 21 cases per 100,000 population) and varies by country/region or study period [1,16]. 

This study using the national insurance database demonstrated an incidence rate of 3.68 per 100,000 population of systemic candidiasis in Taiwan. There are few studies reporting the nationwide burden of candidemia in Asia. In Malaysia and Vietnam, it was estimated to be five per 100,000 population, without any direct data to support these estimates [12,13]. 

Invasive candidiasis is mainly healthcare-associated [15,17]. Multistate point-prevalence survey in USA in 2011 showed that *Candida* species, as a whole, were the leading pathogens causing healthcare-associated bloodstream infection (22%) in acute care hospitals [18]. According to the national surveillance of healthcare-associated infections in the ICUs in 2015, *Candida* species contributed 12% of bloodstream infections in Taiwan and 13% in Korea [19]. Up to 39.4% of systemic candidiasis cases in the Taiwan NHIRD in 2013 occurred in patients who are discharged from ICUs. A multicenter study in Asia in 2011 showed that 23.1% of candidemia occurred in ICU patients [15]. The same study showed that the incidence of candidemia in ICU patients was ten-fold that of all hospitalized patients and the ICU/ total bed ratio affected the incidences of candidemia significantly [15]. The incidence of invasive candidiasis is underestimated if based on blood culture positive cases only as the pooled culture positivity percentage in patients with proven or probable invasive candidiasis is 38% [20,21,22].

Esophageal candidiasis in Taiwan was 6.16 per 100,000 population, while that in Malyasia, Nepal, and Vietnam was estimated to be 19, 10.8, and 36 per 100,000 population, respectively [12,13,14]. Data on estimated annual cases and total burden of selected fungal diseases from other Asia countries were listed in Table 1. 

This study estimated an incidence rate of 0.98 cases of pulmonary aspergillosis per 100,000 populations in 2013. A previous study based on inpatient NHIRD data showed a significant increase in incidence from 0.09 per 100,000 population in 2002 to 0.21 in 2011, which was correlated with the more frequent use of the galactomannan test [23]. This estimate was lower than that in other Asian countries (Table 1) [12,13,14,24]. In China, it was estimated that more than 160,000 cases of invasive aspergillosis (IA) occurred annually, and the overall IA infection rate ranged from 0.29% to 14% depending on different underlying comorbidities [24,25]. In our analysis, nearly half of the pulmonary aspergillosis cases occurred in cancer patients. A single center study during 2008 and 2013 in Taiwan showed that 11.3% of 2083 adults with haematological malignancy not given systemic anti-mold prophylaxis had proven, probable or possible IA. The incidence rate was highest in acute myeloid leukemia (13.7 per 100 patient-year) followed by acute lymphocytic leukemia (11.3 per 100 patient-year) [26]. Among adult patients with acute myeloid leukemia who underwent induction chemotherapy without systemic anti-mold prophylaxis during 2004 and 2009 in the same hospital, 34.6% of them had invasive fungal infections (5.7% proven, 5.0% probable, and 23.8% possible cases) [27]. *Aspergillus* contributed 53% of those with confirmed etiologies. This study showed that the presence of invasive fungal infections in induction chemotherapy independently predicted worse survival [27].

Our study shows a very low incidence of ABPA in Taiwan (0.077/100,000). Allergic bronchopulmonary aspergillosis is not always diagnosed, so grossly low estimates are likely. For example, the NHIRD database recorded 50,293 asthma cases in people over the age of 40 years and if (as in China) 2.5% of these people have ABPA [28,29], then an expected 1257 ABPA patients would be expected in this age group, and probably many more in younger people. Overall the burden of all forms of aspergillosis in Taiwan as well as in other Asian countries are very likely underestimated due to limited availability and turn-around-time of the diagnostic tools. Assuming at least 10% of the 69,648 patients with chronic obstructive pulmonary disease (COPD) are admitted to hospital annually, and that invasive aspergillosis affects 3.9% of these [30], we would anticipate 2271 such cases. A recent surveillance of mycology laboratories in seven Asian countries showed that galactomannan antigen testing is available in 22.8% (55/241) of surveyed laboratories and it is most commonly performed 1–2 times weekly (21/32, 65.6%) [31]. In a 10-year nationwide population study including 407 patients with invasive pulmonary aspergillosis in Taiwan, the reported in-hospital mortality rate was 30.2% [32]. Chronic pulmonary aspergillosis was probably barely diagnosed at all, and certainly not captured by ICD-9-CM, as there was no code for it.

In Taiwan, opportunistic fungal infection in HIV-infected patients is decreasing due to early detection and timely effective antiretroviral therapy [6,33,34]. For example, there was no invasive aspergillosis diagnosed in HIV-infected patients with good compliance of effective antiretroviral therapy in the past decade [34]. Nevertheless, this study showed that HIV-infected patients contributed half of PCP incident cases (50.4%) in Taiwan. In a five-year survey including 3655 HIV-infected patients in Taiwan, the proportion of late ART initiators at risk of PCP (defined as patients with CD4 count <200 cells/mm^3^) decreased from 49.1% in 2012 to 29.0% in 2016 [35]. 

Regarding cryptococcosis, this study provided data for cryptococcal meningitis and enabled comparison with other Asian countries. Based on the NHIRD the incidence rate of cryptococcosis (ICD9 117.5) was 2.87 cases per 100,000 population in 2012 [36] and was 1.04 cases per 100,000 population for cryptoccocal meningitis (this study), which was lower than 2.8 cases per 100,000 population in Malaysia [16] and higher than 0.15 cases in Vietnam [15] (Table 1). This study showed 21% of these incident cryptococcal meningitis cases were in HIV-infected people concordant with our previous nationwide molecular epidemiological study. In that study 54 (24.6%) of 219 patients with culture-confirmed cryptococcosis during 1997-2010 were HIV-infected and only four (1.8%) were solid organ transplant recipients [37]. Another study using the Taiwan NHIRD reported that the incidence of cryptococcal meningitis was stable during 2000 to 2007, and most cases were non-HIV related [38]. Studies have demonstrated the increase in numbers of cryptococcosis in patients other than HIV-infected or solid organ transplant recipients [39,40,41]. Thus, further studies focusing on these patient groups are warranted.

The strength of this study using the nationwide health insurance database for disease burden estimates was its large sample size that comprised nearly all the population. Nevertheless, there are limitations. First, the ICD-9 code for each fungal disease or underlying disease in the database was not validated. Second, the ICD-9 code 484.6 represented “pneumonia in aspergillosis,” which might be either acute or chronic pulmonary aspergillosis. The ICD-9 code was released in 1979; the current version is ICD-11, released in 2018, and extensively revised for fungal disease classification. Third, fungal diseases are very likely underestimated in this study as only five diagnoses for each hospitalization were provided in NHIRD. Moreover, this study did not provide data for urinary tract and cutaneous infections which were probably even more common. The Taiwan national surveillance showed that *Candida* species contributed 31% of pathogens causing healthcare-associated urinary tract infection in the ICUs [19]. Table 2 compares the strengths and limitations of using the health Insurance database for disease burden estimates and hospital-based epidemiological studies.

In conclusion, about 80,000 cases of selected fungal diseases per year within the 23.4 million Taiwanese people were documented by the NHIRD database. Some notable gaps existed, especially for invasive, chronic and allergic aspergillosis, which represents a function of limited awareness and diagnostic use. For less common fungal infections, data are still very limited, and a national reporting system may be considered to achieve better understanding of the burden of fungal diseases in Taiwan. 

## Figures and Tables

**Table 1:** The estimated annual incident case numbers and incidence rates of selected fungal diseases in 2013 in Taiwan and those in other Asian countries.

Fungal Disease		Taiwan, This Study **		Vietnam [12]		Malaysia [13]		Nepal [14]	
	ICD-9 Code	Incident Case Number	Incidence Rate	Incident Case Number	Incidence Rate	Incident Case Number	Incidence Rate	Incident Case Number	Incidence Rate
**Yeasts**									
***Candida* vulvovaginitis**	112.1	45,291	477.05 ^+^	-	-	-	-	-	-
**Recurrent (>4 times/year)**	112.1	9363	98.62 ^+^	1,767,581	3893 *	501,138	4800 ^+^	443,237	2908 ^+^
**Oral candidiasis**	112	21,066	90.13	-	-	-	-	-	-
***Candida* esophagitis**	112.84	1440	6.16	33,107	36	5850	19	2950	10.8
**Systemic candidiasis**	112.5	861	3.68	4540	5	1533	5	-	-
***Candida* peritonitis**	112.85	27	0.12	-	-	230	0.8	-	-
**Pneumocystis pneumonia**	136.3	1251	5.35	608	0.67	1286	4.2	990	3.6
**Cryptococcal meningitis**	321.0	243	1.04	140	0.15	885	2.8	164	0.6
**Molds**									
**Aspergillosis**	117.3, 484.6	567	2.43	14,523	15.99	1018	3.3	1119	4
**Pulmonary aspergillosis (PA)**	484.6	228	0.98						
**PA post Tuberculosis** **(incidence)**	484.6, 010–0.18	60	0.26	-	-	-	-	-	-
**PA post Tuberculosis** **(prevalence)**	484.6, 010–0.18	75 *	0.32 *	-	-	-	-	-	-
**Chronic pulmonary aspergillosis**	None ^#^	-	-	55,509	61	-	-	6611	24.2
**Allergic bronchopulmonary aspergillosis (ABPA)**	518.6	45 *	0.19 *	23,607	26 *	30,062	98	9546	35
**Severe asthma with fungal sensitization**		-	-	31,161	34	39,682	130	12,600	46.1
**Mucormycosis**	117.7	66	0.28	109	0.12	-	-	55	0.2

* Prevalence. Otherwise the table presents the annual incidence rate (cases per 100,000 population). ^+^ Rate of recurrent vaginal candidiasis is per 100,000 adult females. ** Taiwan data was estimated using the nationwide health Insurance database, while the study of Vietnam and Nepal were based on published papers and global data; the study of Malaysia derived its estimation from worldwide data and was based on the methodology of the Leading International Fungal Education (LIFE) program. ^#^ Chronic pulmonary aspergillosis is now coded in ICD11.

**Table 2:** Strengths and limitations of using health Insurance database and published hospital-based epidemiological studies for estimation of national burdens of infectious diseases.

	Strengths	Limitations
National health insurance database	Large sample size Feasible for long term follow up study Feasible for less common disease Comprehensive coverage of various patient groups Time- and cost-saving	Coding bias generated due to reimbursement or other logistic consideration Uncertain quality of diagnosis of underlying diseases or comorbidities in the absence of coding verification Suboptimal accuracy of the diagnosis of infectious diseases in the absence of case definition consensus Missing information regarding detailed time sequence of event (infectious diseases) and risk factors (surgical procedure or other healthcare factors) due to data structure Incomplete data due to no specific ICD9 code for selected fungal pathogens, such as talaromycosis
Hospital-based epidemiological studies	Better quality due to study design, case definition and structured data collection form Detailed time sequence of events (infectious diseases), risk factors (surgical procedure or other healthcare factors) and treatment response	Limited sample size Selection bias due to the characteristics of participating hospitals Time-consuming and resource-dependent

## Supplementary Materials

The following are available online at https://www.mdpi.com/2309-608X/5/3/78/s1, Table S1: ICD-9-CM Coding Algorithms for Charlson Comorbidities [12].
