# Supplementary material for: Fungal Diseases in Taiwan—National Insurance Data and Estimation
Source: J Fungi (Basel). 2019 Aug 21;5(3):78. doi: 10.3390/jof5030078 (PMC6787579; doi:10.3390/jof5030078)
Supplement: Supplementary file 1 [file jof-05-00078-s001.pdf]

Supplementary Table S1. ICD-9-CM Coding Algorithms for Charlson Comorbidities [11].

Comorbidities	ICD-9-CM
Myocardial infarction	410.x, 412.x
Congestive Heart failure	428.x
Peripheral vascular disease	443.9, 441.x, 785.4, V43.4 Procedure 38.48
Cerebrovascular disease	430.x–438.x
Dementia	290.x
Chronic pulmonary disease	490.x–505.x, 506.4
Rheumatic	710.0, 710.1, 710.4, 714.0–714.2, 714.81, 725.x
Peptic ulcer disease	531.x–534.x
Mild liver disease (include 571.2 Alcoholic cirrhosis of liver 571.4 Chronic hepatitis 571.5 Cirrhosis of liver without mention of alcohol 571.6 Biliary cirrhosis)	571.2, 571.4–571.6
Moderate or severe liver disease	456.0–456.21, 572.2–572.8
Diabetes without chronic complication	250.0–250.3, 250.7
Diabetes with chronic complication	250.4–250.6
Hemiplegia or paraplegia	344.1, 342.x
Renal disease	582.x, 583–583.7, 585.x, 586.x, 588.x
Malignancy excluding neoplasm of lymphatic and hematopoietic tissue and excluding malignant neoplasm of skin	140.x–172.x, 174.x.–195.8,
Neoplasm of lymphatic and hematopoietic tissue	200.x–208.x
Metastatic solid tumor	196.x–199.1
AIDS/HIV	042.0-044.0, V08
Organ or tissue replaced by transplant	Procedure 55.69, 50.59, 37.51, 33.50, 33.51, 33.52, 33.6, 52.80, 52.86, 46.97, Diagnosis V42.0, 42.1, 42.6, 42.7
